# Agro-ecology, household economics and malaria in Uganda: empirical correlations between agricultural and health outcomes

**DOI:** 10.1186/1475-2875-13-251

**Published:** 2014-07-03

**Authors:** Benjamin Wielgosz, Edward Kato, Claudia Ringler

**Affiliations:** 1 World Economic Forum, 91-93 route de la Capite, 1223 Cologny, Geneva, Switzerland; 2 International Food Policy Research Institute, 2033 K Street, NW, Washington, DC 20006-1002, USA

**Keywords:** Uganda, Agriculture, Environmental management, Integrated vector management, Crop choice, Pesticide resistance

## Abstract

**Background:**

This paper establishes empirical evidence relating the agriculture and health sectors in Uganda. The analysis explores linkages between agricultural management, malaria and implications for improving community health outcomes in rural Uganda. The goal of this exploratory work is to expand the evidence-base for collaboration between the agricultural and health sectors in Uganda.

**Methods:**

The paper presents an analysis of data from the 2006 Uganda National Household Survey using a parametric multivariate Two-Limit Tobit model to identify correlations between agro-ecological variables including geographically joined daily seasonal precipitation records and household level malaria risk. The analysis of agricultural and environmental factors as they affect household malaria rates, disaggregated by age-group, is inspired by a complimentary review of existing agricultural malaria literature indicating a gap in evidence with respect to agricultural management as a form of malaria vector management. Crop choices and agricultural management practices may contribute to vector control through the simultaneous effects of reducing malaria transmission, improving housing and nutrition through income gains, and reducing insecticide resistance in both malaria vectors and agricultural pests.

**Results:**

The econometric results show the existence of statistically significant correlations between crops, such as sweet potatoes/yams, beans, millet and sorghum, with household malaria risk. Local environmental factors are also influential- daily maximum temperature is negatively correlated with malaria, while daily minimum temperature is positively correlated with malaria, confirming trends in the broader literature are applicable to the Ugandan context.

**Conclusions:**

Although not necessarily causative, the findings provide sufficient evidence to warrant purposefully designed work to test for agriculture health causation in vector management. A key constraint to modeling the agricultural basis of malaria transmission is the lack of data integrating both the health and agricultural information necessary to satisfy the differing methodologies used by the two sectors. A national platform for collaboration between the agricultural and health sectors could help align programs to achieve better measurements of agricultural interactions with vector reproduction and evaluate the potential for agricultural policy and programs to support rural malaria control.

## Background

Roughly 250 million people are currently infected each year by malaria parasites and more than three billion are at risk for infection [1,2]. Despite a century of scientific progress in preventing, treating, and understanding the parasite and its means of reproduction, malaria continues to impose one of the world’s heaviest burdens of disease in terms of disability adjusted life years (DALYs) globally [3].

The burden of malaria is concentrated in sub-Saharan Africa and disproportionately affects the rural poor there who are primarily engaged in agricultural production for their livelihoods. Uganda is an important case study because it is representative of the challenges facing malaria control in Africa. Malaria is endemic in over 95% of the country and is the leading cause of morbidity and mortality in Uganda [4]. The baseline transmission potential in Uganda has likely been increasing over the last twenty to forty years due to deforestation, road construction, proliferation of borrow-pits, and wetland cultivation [5,6].

It is not possible using the current set of conventional interventions to eliminate malaria from such high transmission areas [7,8]. Current evaluations of insecticide-treated bed nets (ITNs), artemisinin combination therapy (ACT), and indoor residual spraying (IRS) suggest that while these interventions produce dramatic declines in infection, morbidity, and mortality, the declines are not sufficient to interrupt transmission, particularly in rural, agricultural areas of Africa. Even with the scaling-up of conventional interventions to high coverage rates for sustained periods, transmission will still persist [9]. The WHO estimates that 30 to 53 percent of the global malaria burden (half a million deaths) is attributable to *modifiable* environmental factors [10].

### Agricultural pathways to vector reproduction

Agriculture is the primary means by which rural populations manage and modify their environment which makes it an especially important strategic element of rural malaria control. The constraints on conventional malaria control policies in high-transmission settings such as Uganda make it important to explore the potential for agricultural as a form of environmental management to contribute to reducing baseline transmission of malaria in Uganda. The development and spread of insecticide resistance in anopheles mosquito populations across the globe [11] adds to the urgency of cooperation between the agricultural and health sectors on this issue as the gains from conventional interventions may be threatened without improved interdisciplinary coordination.

The interactions between agricultural systems and malaria have been documented and studied extensively [1] but the primary consideration throughout this topic is how crop, land and water management implemented in agriculture can alter the local reproductive rate of malaria transmitting mosquitoes or their ecological competitors, fostering or reducing malaria transmission in rural populations. In the case of Uganda, cultivation of maize, rice, cotton, and a variety of tree crops have been identified as potentially impacting rural malaria transmission for varying reasons.

Maize is the most widely cultivated crop in Uganda and may contribute to vector reproduction as its pollen is a food source for the larvae of anopheles vectors in Africa [12]. Cotton by comparison has a robust history of interaction with malaria vectors based on the heavy agricultural pesticide-use associated with the crop which may lead to local insecticide resistance [13].

Rice is a crop of particular interest for malaria control due to the potential for interaction between irrigation systems and mosquito reproduction. However, large numbers of mosquitoes do not necessarily coincide with higher malaria incidence. Irrigation systems may favour mosquito species that do not transmit malaria over those that do, referred to as the “paddies paradox”. Other analyses [14] find that development of rice irrigation programmes in areas of stable malaria can be associated with a decline in the prevalence of malaria in rice-growing communities compared with non-rice-growing areas due to improved socioeconomic status and higher adoption of anti-malaria protection measures.

Tree-Crops may also play a role in anopheles reproduction. Many species of anopheles mosquitoes have been documented as adapting based on local agroforestry patterns [15]. In some cases [16] the *Anopheles gambiae* uses tree holes in specifically in acacia, avocado, and mango trees as breeding sites. While a particular crop may favour or disfavour reproduction, the majority of the environment in agricultural systems tends to be in various uncultivated forms and there are a plethora of studies on the links between differing land-cover types and malaria vector reproduction [17,18]. Land uses such as pasture for livestock, fallowing of plots during periods, and surrounding areas of bush can all influence the ecology of mosquito populations.

This limited set of farm level examples already capture a the diverse range of ecological pathways by which agricultural management can impact vector reproduction by affording mosquito populations with varying levels of larval food, pesticide exposure, standing water, sunlight, protection from predation, and alternative blood-meal sources such as cattle or pigs. This ecological complexity is both at the heart of agricultural impacts on malaria transmission and also the deepest challenge to successful collaboration between the agricultural and health sectors.

## Methods

### Data and statistical methodology

The analysis of household malaria is based on geo-referenced data from the 2005–2006 Uganda National Household Survey [19] which integrates agricultural production, socio-economic and health information on household members. This dataset is one of the few available which contains both agricultural and health modules along with the geographic coordinates required to also include local environmental factors.

The UNHS survey was designed to answer a range of nationally important questions by surveying 42,228 individuals in 7,426 households across Uganda. Every individual was asked to self-report malaria: “Have you had malaria in the past 30 days?” Diagnostic testing for malaria would be a far more accurate measure of malaria, but this limitation is common to the datasets available which include agricultural and socioeconomic information. Because these explanatory variables of interest function at the household level, the primary dependent variable is constructed from the original dataset as the proportion of individuals in each household who experienced malaria in the past 30-days.

Malaria risk is not evenly distributed across all age groups. Children and those with compromised immune systems such as the elderly or HIV-positive are particularly susceptible to malaria. One of the most common methods of measuring malaria transmission is to evaluate the prevalence of parasite in the spleens of children under the age of ten who may not yet have acquired immunity to the parasite through previous exposure [9].

To evaluate age-dependent malaria response as well as control for the confounding of demographic factors with malaria correlations, the full UNHS sample of households was broken down into subsets of those containing specific age-ranges within the household (Table 1). The individual malaria responses were aggregated within each household based on the following age groups: 0 to 4, 5 to 9, 10 to 19, 20 to 39, 40, to 59, and 60 or more years of age. A subset for the 0 to 4 age group would be all those households with 0 to 4 year old children. For each household a total malaria rate was calculated as well as a rate within each age-group (the proportion of individuals with malaria in each age group out of the total number of household members in that age group). Results are presented to explain both the total household malaria proportions (using the full UNHS sample) as well as the proportions for each age group’s sub-sample.

**Table 1:** Sample subsets

**Age-group reporting malaria**	**Size**	**% of households**
Full sample	7369	100%
Age 0 to 4	4309	58%
Age 5 to 9	3999	54%
Age 10 to 19	4680	64%
Age 20 to 39	5759	78%
Age 40 to 59	2938	40%
Age 60 plus	1428	19%

5718 Households reported data for agriculturally cultivated plots.

In addition to controlling for demographic effects, the socio-economic status of households was integrated using categorical control variables based on the annual consumption expenditure for each household. Households were categorized into three terciles based on these annual expenses. The poorest tercile was determined as 0 to 72,000 Ugandan shillings (UGX), the median tercile as 72,000 to 245,000 UGX, and the wealthiest as 245,000 to 28 million UGX. These correspond to ranges of $0 – 40, $40 – 135, and $135 – 15,000 in 2005 US dollars. The inclusion of these wealth terciles as control variables was essential to address the interactions and frequent codependence between socio-economic status and health outcomes typical at the household level. Annual consumption expenditure terciles were calculated without including expenditure on healthcare which was totaled separately in the UNHS health module and used to construct a distinct 30-day health expenditure variable for inclusion in the Tobit analyses.

### Explanatory variables

The selection and in some cases construction of the independent variables was a complex process owing to the nature of the suggested relationships involved. In addition to household demographics and socio-economic status based on consumption, the gender of the head of household (with 75 percent of households male-headed and 25 percent of households female-headed) was selected as a key household variable along with the literacy and age of the head of household in order to provide a more complete profile of social factors influencing health outcomes and reduce the interaction between socio-economic status and particular crop choices that could reflect that status.

As the dependent variable of interest (proportion of household members experiencing malaria) is a health outcome, both 30 Day Health Expenditure (UGX) and bed net useage in the past 30 days were included to account for differing health status and behaviour between households. Respondents to the UNHS were asked which household members slept under a bed net during the past 30 days. As a household level variable, bed net usage was constructed similarly to malaria, as a proportion of all household members or as a proportion for each age group (Table 2).

**Table 2:** Descriptive statistics

	**Variable**	**Observations**	**Mean**	**Standard deviation**	**Min**	**Max**
Dependent variables	(All ages) % reporting Malaria	7369	0.15023	0.21620	0	1
	(Age 0 to 4) % reporting Malaria	4309	0.23315	0.37210	0	1
	(Age 5 to 9) % reporting Malaria	3999	0.13815	0.30436	0	1
	(Age 10 to 19) % reporting Malaria	4680	0.10270	0.24741	0	1
	(Age 20 to 39) % reporting Malaria	5759	0.13291	0.29118	0	1
	(Age 40 to 59) % reporting Malaria	2938	0.14875	0.33512	0	1
	(Age 60 plus) % reporting Malaria	1428	0.17892	0.37054	0	1
Socio economic & health variables	Head of Household - Gender	7369	0.72819	0.44492	0	1
	Head of Household - Age	7369	42.18619	15.69120	13	105
	Head of Household - Literacy	7369	0.41254	0.49232	0	1
	Household - Urban	7369	0.22798	0.41956	0	1
	Household Consumption Tercile - Poorest	7369	0.33790	0.47303	0	1
	Household Consumption Tercile - Middle	7369	0.33193	0.47094	0	1
	Household Consumption Tercile - Wealthiest	7369	0.32990	0.47021	0	1
	Household Health Expenditure	7369	13,272	34,342	0	955,000
	(past 30 days)					
	(All ages) % using bed net	7369	0.18624	0.33987	0	1
	(Age 0 to 4) % using bed net	4309	0.21257	0.39745	0	1
	(Age 5 to 9) % using bed net	3999	0.13125	0.33269	0	1
	(Age 10 to 19) % using bed net	4680	0.11964	0.30756	0	1
	(Age 20 to 39) % using bed net	5759	0.23628	0.40772	0	1
	(Age 40 to 59) % using bed net	2938	0.21894	0.40815	0	1
	(Age 60 plus) % using bed net	1428	0.15710	0.35977	0	1
Agricultural variables	Total Precipitation	7369	259.19560	108.43070	0	607.46790
	(past 90 days)					
	Average Maximum Temperature	7369	29.19034	2.35618	22.60880	37.41636
	(past 90 days)					
	Average Minimum Temperature	7369	17.97333	1.71088	12.43053	22.47368
	(past 90 days)					
	Minutes Waiting at Water-source	7369	54.44728	67.60944	0	1,500
	Distance to Water-source from homestead	7369	1.56396	4.58784	0	100
	Livestock - Number of Chickens	7369	13.59520	47.84819	0	1,259
	Livestock - Number of Cattle	7369	5.69982	21.62892	0	555
	Livestock - Number of Pigs	7369	2.21116	46.63361	0	2,000
	Agricultural Extension Visit in the past 12 months	7369	0.16827	1.10013	0	36
	Crop Protection Information	7369	0.30805	0.46172	0	1
	Disease Control Information	7369	0.33614	0.47242	0	1
	Pesticide Application Area	7369	0.10920	1.25976	0	80.93726

### Agricultural variables

In order to compare the effects of different crop cultivation choices by farmers, a set of crop-choice categories and a distinct scale variable for the total crop area cultivated by each household were constructed from the plot-level data. Crop Choice variables are modelled as proportions of total crop areas reported for each agricultural household. Maize is used as the reference category, such that the included crop-choices indicate positive or negative malaria interactions relative to maize. Maize, Pasture, Bush, Bananas, Fallow, Cassava, Beans, Sweet Potato/Yam, Coffee, Sorghum, Groundnuts, and Millet were selected based on their status as the largest cultivation areas within the sample.

In addition to the most widely cultivated crops, some crop choices were included based on the established literature on malaria and agriculture. Rice was selected for inclusion based on its relationship to irrigation. Tree Crops are included as an aggregated category despite their relatively small cultivated areas due to their potential role in vector reproduction (tea, cocoa, pineapple, passion fruit, jackfruit, avocado, mango, paw-paw, oranges, oil palm, and acacia). Plot areas enumerated as pasture, fallow, and bush are included as crop-choice categories to provide a comparison of un-planted land-use. Finally, ‘Other Crops’ was used as an aggregated category in order to complete the proportional choice model (Table 3).

**Table 3:** Crop statistics

**Crop-choice category**	**Crop**	**Number of households cultivating crop**	**Total area in sample (Ha)**
**Maize**	**Maize**	**3808**	**1223.38**
Pasture	Pasture	283	1396.18
Bush	Bush	439	982.76
Bananas	Bananas	3066	981.78
Fallow	Fallow	1143	885.45
Cassava	Cassava	3512	879.63
Beans	Beans	3057	605.63
Sweet Potato/Yam	Sweet Potato/Yam	2965	491.33
Coffee	Coffee	1705	414.68
Sorghum	Sorghum	1092	298.12
Groundnuts	Groundnuts	1333	242.64
Millet	Millet	790	202.09
Rice	Rice	262	95.89

Cotton was not included as an independent crop choice due to its infrequent cultivation in Uganda (it was aggregated within the ‘Other Crops’ category). However, 75 percent of pesticide in Uganda is applied to cotton [20]. For this reason, pesticide application area is included as an agricultural variable rather than cotton as a crop-choice as the key linkage between cotton and malaria is based on pesticide application to begin with. Livestock keeping, distance to water sources, and visitation by agricultural extension workers were also included to test for any potential interactions with vector reproduction or control.

### Seasonal meteorological data

The geographic coordinates at the household level are a strength of the UNHS dataset as they allow for the integration of high quality environmental data into the analysis. As the national household survey was conducted over the course of almost 18 months of data collection, capturing the seasonal variation of malaria transmission was essential. Availability of water-sources and temperatures may affect the reproduction of both the anopheles mosquitoes that transmit malaria parasites, and the reproduction of the parasites themselves in the mosquitoes. The date of the interview was used in combination with daily gridded precipitation and temperature data to capture both seasonal and geographic variation in water availability and local temperature for each household.

Malaria parasites can only reproduce in the mosquito stage when the temperature is above 14°C [21]. Many studies continue to confirm a significant positive relationship between malaria and increased daily temperature minimums but a negative relationship with increased daily maximum temperatures [22], with optimal transmission potentially occurring at temperatures in the 32–33°C range [23]. Analyses in the East African highlands have [24] ranked risk factors for malaria and found that monthly rainfall and minimum temperatures were the top environmental predictors of malaria risk, reinforcing the conclusions of other models [25,26] that minimum temperatures are a primary constraint on transmission.

In order to integrate seasonal temperature and precipitation values into the model, a time-frame for these climatic effects was determined based on the life-cycles of the mosquito and parasite. Anopheles mosquitoes have four stages in their life cycle: egg, larva, pupa, and adult. The first three developmental stages take place in water and occur over 5–14 days. The adult female that emerges generally lives for 14 to 30 days during which the malaria parasite may complete the “extrinsic” portion of its life cycle in the mosquito over the course of 10 to 18 of those days [27]. Symptoms usually appear 7 to 15 days after an infective mosquito bite [28]. Taken in sequence, this indicates that the time between the laying of a mosquito egg in water and the experience of malaria symptoms in an infected individual may be up to 60 days. Respondents to the UNHS 2005/06 household survey were asked to self-report malaria incidence occurring in the 30 days preceding the interview which in combination with the interview date provide a reasonable estimate of the 90 days in 2005 to 2006 that a household’s malaria risk would be affected by temperature and precipitation.

The 90-day period preceding the household interview date form the basis for aggregating daily precipitation and averaging daily temperature data from the Agricultural Model Intercomparison and Improvement Project (AgMIP) which is a high resolution time series weather data product of the NASA Goddard Institute for Space Studies developed for the Coordinated Climate-Crop Modelling Project (C3MP) (Ruane and Goldberg, Personal Communication). The AgMIP climate product integrates the best available local data from weather stations with NASA weather satellite observations to interpolate the most reliable possible weather series each 15-minutes geographic grid cell (roughly 27.6 km by 27.6 km close to the equator). GPS coordinates for households were re-projected from decimal degrees into the ESRI Africa Equidistant Conic map-projection. These coordinates were then used to join the 15-minute gridded AgMIP daily precipitation and temperature aggregations and averages. Three meteorological variables were generated from daily weather data: the total precipitation over the previous 90 days, the average minimum daily temperature over the previous 90 days, and average maximum daily temperature over the previous 90 days.

### Model selection

As the dependent variable being modelled is a proportion, Tobit regressions are used to model agriculture, socio-economic, and other independent variables as they affect the household malaria rates (by age-group). Due to the nature of the dependent variable expressed as a proportion of household members or proportion of each age group within the household, using an Ordinary Least Squares (OLS) estimator would lead to biased and inconsistent estimates because of the censored nature of the dependent variables. A censored regression Tobit model [29] accounts for both the left and right censoring in the dependent variables. All the models are estimated in STATA version 12 using a Two-Limit Tobit model with the lower censoring at 0 for households where no members report malaria and with upper censoring limit at 1 for households where all members report malaria.

### Endogeneity and covariance

The variables of interest are the different crop-choices practiced by the household, and joined weather data, but because the dataset is cross-sectional, crop choices and other agricultural variables are likely to be endogenous to the household and this may lead to an endogeneity bias in the estimates. The conventional approach of correcting for endogeneity is to use an instrumental variables approach that requires a set of excluded instruments to instrument for crop choice variables. A rich set of instruments was applied to the tobit models consisting of plot investments, land management and plot tenure conditions. However like in many maximum likelihood estimations, the instrumental variable tobit (IV-tobit) failed to converge after several trials and fixes. The results of the instrumental tobit is therefore not reported in this paper but rather the ordinary tobit estimates.

The failure of converge using various instrumenting variables placed a major limitation on the analysis by preventing any claim of causation among the independent variables.

Despite the failure of the IV-tobit, estimating the tobit as a linear two-stage least squares model was conducted to consider if endogeneity affects the results in the ordinary tobit model. Interestingly, the results of the tobit and the linear two-stage least squares model to be qualitatively similar which can provide confidence in the robustness of the findings. The direction of correlations was consistent between the tobit models estimated and two stage least squares model. This should imply that any endogeneity affecting the magnitude of the correlation coefficients, does not have any impact on the sign or direction of those coefficients.

A covariance matrix was used to test for multicollinearity. Multiple correlation among the explanatory variables (which reduces statistical power) was not a serious problem as revealed by the low correlation coefficients from the correlation matrix. All standard errors in the regressions are adjusted for heteroscedasticity using the White-Huber correction [30]. This provides confidence in the independence of the explanatory variables.

## Results and discussion

### Key socio-economic and health findings

The results of the tobit analysis were relatively in line with public health literature on malaria in Uganda, although in some cases it is likely that the self-reporting of malaria undermined what would otherwise have been stronger correlations between explanatory variables and household malaria risk. In the total sample, both wealthier and poorer households were associated with less malaria than the middle wealth tercile. This effect was not seen consistently across age groups, but was observed in the five to nine age group.

The gender of the head of household was statistically significant in the total-sample and every age group except for adults age 20 to 39. A notable detail is that the association was negative (meaning that male headed households reported lower malaria prevalence than female-headed households) except for the children under-five age group where female-headed households reported lower malaria prevalence among these children. This may have interesting implications for reducing child mortality due to malaria depending on the explanation.

Urban households were associated with less malaria compared to rural households in the complete sample. In the age-disaggregated analyses, this effect was only observed in the 10 to 19 year old age group. Urban areas usually have much better access to health services. In high transmission areas such as central and northwestern Uganda urban areas have been shown to have 18 percent lower malaria prevalence than rural areas [31]. The analysis presented in this paper supports the urban rural trend identified in youth for Uganda with larger magnitude coefficients for younger age groups (Table 4).

**Table 4:** Socio-economic and health determinants of malaria proportion Tobit by age-group

**Socio economic & health variables**	**All ages**		**0 to 4**		**5 to 9**		**10 to 19**		**20 to 39**		**40 to 59**		**60 plus**	
	**Coef**	**P>|t|**	**Coef**	**P>|t|**	**Coef**	**P>|t|**	**Coef**	**P>|t|**	**Coef**	**P>|t|**	**Coef**	**P>|t|**	**Coef**	**P>|t|**
Head of Household (Gender)	***-***	* 0.0580	***+***	** 0.0400	***-***	*** 0.0000	***-***	*** 0.0010		0.6220	***-***	* 0.0920	***-***	** 0.0200
Head of Household (Age)	***-***	** 0.0490		0.7420		0.7290		0.8670	***-***	*** 0.0000		0.3600		0.3700
Head of Household (Literacy)	***+***	* 0.0840		0.2010		0.3510		0.6020	***+***	* 0.0820	***+***	** 0.0320		0.4320
Household (Urban)	***-***	*** 0.0100		0.1810		0.8110	***-***	* 0.0590		0.2100		0.6810		0.5660
Household Consumption Tercile (Poorest)	***-***	** 0.0190		0.4280	***-***	** 0.0210		0.3880		0.5570		0.3270	***-***	* 0.0760
Household Consumption Tercile (Middle)		*Omitted for Categorical Comparison*												
Household Consumption Tercile (Wealthiest)	***-***	* 0.0780		0.7960	***-***	*** 0.0100		0.3410		0.8880		0.1820		0.1500
Household Health Expenditure (past 30 days)	***+***	*** 0.0000	***+***	** 0.0350	***+***	*** 0.0030	***+***	*** 0.0000	***+***	*** 0.0000	***+***	** 0.0130		0.3170
% of Age Group using Bed Net		0.6620	***+***	*** 0.0030	***+***	*** 0.0060		0.4980		0.2290		0.6510		0.5990

*P < 0.05.

**P < 0.01.

***P < 0.001.

+A positive correlation between malaria risk and the explanatory variable.

-A negative correlation between malaria risk and the explanatory variable.

Health expenses were consistently associated with malaria cases and in the total sample and across all age groups except for the 60 and older age group. This suggests that almost all households in Uganda are spending income on malaria treatment when the disease is diagnosed or suspected. Across all wealth terciles, those households reporting malaria cases spent roughly 60 percent more on health expenses in the past 30 days than households reporting no malaria (Table 5). Although this increase in health expenses was similar across wealth terciles, this increase in health expenses exacerbated the relatively larger proportion of monthly consumption that poorer and middle terciles devote to health related expenses. While in wealthier households malaria treatment might increase health expenses from 14 to 24 percent of a monthly budget, in the poorer tercile a malaria case would increase monthly health expenses from 61 to 70 percent of monthly cash expenditures (Table 6). Any reduction in malaria prevalence would, therefore, improve household budgets and even a cost effective programme of malaria education to reduce misdiagnosis might have a significant financial benefit to participating households.

**Table 5:** Average of 30 day health expenditure (UGX)

	**Poorer tercile**	**Middle tercile**	**Wealthy tercile**	**Total sample**
Malaria in past 30 days	7,219	13,785	29,293	17,354
No Malaria	4,155	8,131	17,839	9,620

**Table 6:** 30 day health expenditure as % of monthly consumption including health expenses

	**Poorer tercile**	**Middle tercile**	**Wealthy tercile**	**Total sample**
Malaria in past 30 days	70%	54%	24%	32%
No Malaria	61%	42%	14%	20%

Bet net use was positively correlated with malaria prevalence for children under-5 and children under-10. This likely represents a reverse-causality where parents are acting to protect children using this heavily promoted intervention when the children are known to be at high risk of malaria infection. Where children have been taken for malaria treatment in the past 30-days, there is a good chance that families were provided bed nets at the time of treatment to prevent recurrence in the future.

### Agro-ecological associations

There is a high degree of covariance between total precipitation, average maximum temperature, and average minimum temperature during the past 90 days (Table 7). In the total sample and all of the age groups younger than 40, maximum daily temperature was negatively correlated with malaria prevalence. This is consistent with the literature review [1] which found negative associations with increased daily maximum temperatures [22] and likely indicates that higher temperatures are inhibiting parasite reproduction in the Ugandan context. The positive correlation between daily minimum temperatures observed in the 5 to 9 age group is also consistent with the literature [24,25].

**Table 7:** Co-variance of AgMIP meteorological variables

	**Total precipitation (past 90 days)**	**Average maximum temperature (past 90 days)**	**Average minimum temperature (past 90 days)**
Total Precipitation (past 90 days)	1.0000		
Average Maximum Temperature (past 90 days)	−0.3177	1.0000	
Average Minimum Temperature (past 90 days)	0.1333	0.5748	1.0000

The agricultural variables relating to water-collection, livestock, and agricultural extension information did not demonstrate significant correlations with malaria prevalence. The exception to this was visitation by agricultural extension workers in the past 12 months, which demonstrated weak association with malaria prevalence in older age groups. No association was detected between malaria and pesticide application in the analyses (Table 8).

**Table 8:** Signs of agricultural determinants of malaria proportion Tobit by age-group

**Agricultural variables**	**All ages**		**0 to 4**		**5 to 9**		**10 to 19**		**20 to 39**		**40 to 59**		**60 plus**	
	**Coef**	**P>|t|**	**Coef**	**P>|t|**	**Coef**	**P>|t|**	**Coef**	**P>|t|**	**Coef**	**P>|t|**	**Coef**	**P>|t|**	**Coef**	**P>|t|**
Total Precipitation (past 90 days)		0.9280		0.9820		0.2200		0.9310		0.5070		0.2360		0.2230
Average Maximum Temperature (past 90 days)	***-***	*** 0.0000	***-***	* 0.0840	***-***	*** 0.0000	***-***	** 0.0190	***-***	*** 0.0080		0.4150		0.7530
Average Minimum Temperature (past 90 days)		0.4270		0.2320	***+***	*** 0.0010		0.8750		0.3160		0.4610		0.2350
Minutes Waiting at Water source		0.3270		0.5190		0.2790		0.9460		0.5130		0.3940		0.6460
Distance to Water source from homestead		0.3320		0.3120		0.5550		0.2260		0.1960		0.4250		0.2750
Livestock (Number of Chickens)		0.4230		0.8790		0.3710		0.6380		0.5380		0.5550		0.9660
Livestock (Number of Cattle)		0.1870		0.6800		0.4310		0.7330		0.7900		0.3650		0.6470
Livestock (Number of Pigs)		0.8710		0.9780		0.5510		0.6030		0.5200		0.1010		0.4760
Agricultural Extension Visit in the past 12 months		0.5720		0.1670		0.6430		0.2190	***-***	* 0.0540		0.5540	***+***	* 0.0690
Crop Protection Information		0.1110		0.2430		0.3240		0.7240		0.8170		0.3950		0.8190
Disease Control Information		0.5580		0.7710		0.8580		0.2610		0.8400		0.1130		0.8890
Pesticide Application Area		0.4820		0.1430		0.7100		0.7870		0.1450		0.5110		0.2980

*P < 0.05.

**P < 0.01.

***P < 0.001.

+A positive correlation between malaria risk and the explanatory variable.

-A negative correlation between malaria risk and the explanatory variable.

The crop-categories with statistically significant associations to malaria at the 0.05 significance threshold are the sweet potatoes/yams, beans, millet, and the ‘Other Crops’ categories with consistent coefficient signs making interpretation reasonable. Sweet potatoes/yams, beans and the ‘Other Crops’ categories are consistently associated with higher household malaria prevalence in these Tobit analyses, while millet demonstrated a negative association in comparison to maize. One possible explanation for this, particularly in the case of Sweet potatoes/yams may be due to the furrowed planting rows associated with this crop which are used in conjunction with flooding to keep the plants watered. Essentially, sweet potatoes may increase the breeding environment available to mosquitos as they increase sunlit standing water in the environment. By contrast, millet is generally well drained plots which are not flooded due to the plants lower water requirements.

As beans and ‘other crops’ entail a diverse range of cultivation practices it is more difficult to interpret any explanation for the results. However, these particular results can also be considered inversely as statements with respect to maize, which may have comparatively lower malaria associated with its cultivation in Uganda than sweet potatoes/yams, beans, and the ‘Other Crops’, but more when compared to millet (Table 9).

**Table 9:** Crop-choice determinants of malaria proportion Tobit by age-group

**Crops as a proportion of total crop-area**	**All ages**		**0 to 4**		**5 to 9**		**10 to 19**		**20 to 39**		**40 to 59**		**60 plus**	
	**Coef**	**P>|t|**	**Coef**	**P>|t|**	**Coef**	**P>|t|**	**Coef**	**P>|t|**	**Coef**	**P>|t|**	**Coef**	**P>|t|**	**Coef**	**P>|t|**
Maize	*Omitted for Categorical Comparison*													
Other Crops	***+***	** 0.0180		0.3360		0.3800		0.4770	***+***	* 0.0530		0.3870		0.3030
Bananas		0.7690		0.6890		0.8610		0.9850		0.2130		0.3330		0.8810
Coffee		0.4460		0.1210		0.4950		0.3510		0.6660		0.1970		0.2080
Beans		0.1160		0.8440	***+***	** 0.0160		0.5470		0.1140		0.3470		0.4360
Cassava		0.9390		0.2240		0.1290		0.5110		0.8150		0.9100		0.2140
Groundnuts		0.9700		0.8470		0.8980		0.4930		0.8520		0.5930		0.6370
Sweet Potato / Yam	***+***	** 0.0450		0.2310		0.4870	***+***	* 0.0880		0.6400		0.5010		0.5700
Millet		0.9360	***-***	** 0.0150		0.2460		0.6150		0.4160		0.4390		0.9590
Rice		0.9290		0.5770		0.1410		0.4580		0.8780		0.4550		0.1580
Sorghum		0.8290		0.9050	***+***	* 0.0720		0.9580		0.7290	***-***	** 0.0170		0.6790
Tree Crops		0.8480		0.5970		0.4920	***+***	* 0.0940		0.9010		0.8580		0.1840
Fallow		0.4790		0.4770		0.2480		0.5770		0.2590		0.5590		0.3260
Bush		0.5970		0.4950		0.4340		0.6010		0.7270		0.7840		0.2660
Pasture		0.4910		0.7400		0.3180		0.5510		0.9000		0.1540		0.2080
Parcel Size		0.2030		0.1920		0.4700		0.5740		0.4370		0.4680		0.7780

*P < 0.05.

**P < 0.01.

***P < 0.001.

+A positive correlation between malaria risk and the explanatory variable.

-A negative correlation between malaria risk and the explanatory variable.

### Implications

In spite of numerous technical challenges presented in attempting to model the interactions between agro-ecology, household economics, and malaria transmission, the analyses demonstrate valuable avenues of exploration for future research agendas and programme design. Even without instrumentation or spatial spillover effects included in the model, malaria prevalence is clearly associated (positively or negatively) with a wide variety of crop-choices, as well as socioeconomic and demographic variables.

Wealth, education, gender, and bed net use are clearly associated with malaria at the household level, with malaria risk differing across age groups. Health expenses were consistently associated with malaria cases suggesting household budgetary impact. Urban households were associated with less malaria compared to rural households. Male headed households reported lower malaria prevalence than female headed households except for the children-under-5 age group where female headed households reported lower malaria prevalence.

Daily maximum temperature was negatively correlated with malaria prevalence, while daily minimum temperatures were positively correlated with malaria prevalence. These findings confirm that these trends documented in the broader literature on malaria forecasting are applicable to the local Ugandan context.

Based on the cross-sectional framework, there is associative evidence to justify improved agricultural-health data collaborations to establish causal evidence for interactions between the two sectors. Implementing survey and study designs to establish causal evidence would allow for evidenced-based integration of rural service delivery on the part of agricultural extension and village health teams in Uganda or more appropriate targeting of interventions to specific social and demographic groups.

The problem of non-convergent instrumentation has implications for collaborative data collection between the agricultural and health sectors. Many of the choices in how to model the interactions between agriculture and malaria presented in this paper have been driven by what is possible using available data resources. A key constraint to modeling the agricultural basis of malaria transmission is the lack of data integrating both health and agricultural information necessary to satisfy the differing empirical evidence methodologies used by the two sectors. A dataset designed to explore agricultural-health hypotheses would have to be designed in a radically different method from current health indicator surveys or agricultural-census surveys which tend to focus on health programme monitoring or agronomic productivity, but not basic eco-epidemiology or environmental economics of the type required to quantify malaria transmission.

Such a study would require baseline measurement of malaria prevalence in all children under the age of 10 using rapid diagnostic test rather than self-reporting to eliminate inaccurate measurement of disease outcomes. Similarly, environmental and agricultural factors would need to be measured at the village level rather at the household level to account for local spillover effects between households and farms within mosquito flight distances. Finally, such data collection would have to be planned around seasonal cycles of temperature, rainfall, malaria transmission, crop planting, and other temporal effects integral to annual malaria transmission.

Collaborative implementation would be required drawing on the expertise of agricultural economists, malaria control and surveillance specialists, medical entomologists, and meteorologists. Such interdisciplinary studies are rare due to institutional and financial disincentives, but are also important for the measurement and policy design concerning issues such as malaria and rural poverty where the lack of empirical evidence prevents harmonized cooperation between village health workers and agricultural development officers working in the same communities.

## Competing interests

The authors declare that they have no competing interests.

## Authors’ contributions

BW carried out initial topic scoping, geographic information systems, statistical regressions, literature review. EK carried out the econometric instrumentation, socio-economic and demographic disaggregation design, and statistical methodology review. CR carried out grant application and funding application, analytical and policy framing, agricultural economic analysis, and internal review of article. All authors read and approved the final manuscript.
